# P-87. Evaluation of Antibiotic Prescribing Practices and Outcomes of Vertebral Osteomyelitis

**DOI:** 10.1093/ofid/ofaf695.316

**Published:** 2026-01-11

**Authors:** Kaitlyn Reasoner, Karen C Bloch, Lauren Nicholas S Herrera, Omar Zakieh, Byron Stephens, Zhiguo Zhao, Jim Zhang, Milner Staub

**Affiliations:** Vanderbilt University Medical Center, Nashville, TN; Vanderbilt University Medical Center, Nashville, TN; Vanderbilt University Medical Center, Nashville, TN; Vanderbilt University Medical Center, Nashville, TN; Vanderbilt University Medical Center, Nashville, TN; Vanderbilt University Medical Center, Nashville, TN; Vanderbilt University Medical Center, Nashville, TN; Vanderbilt University Medical Center, VA Tennessee Valley Healthcare System, Nashville, TN

## Abstract

**Background:**

Vertebral osteomyelitis comprises 3% to 5% of osteomyelitis cases, with increasing incidence in the United States. Guidelines recommend antimicrobial therapy for a minimum of 6 weeks and up to 12 weeks. Retrospective studies of native vertebral osteomyelitis demonstrated no significant difference in outcomes in patients who received oral versus intravenous (IV) therapy. Other retrospective reviews have identified multiple factors associated with relapsed infection or adverse outcomes. We analyzed antibiotic prescribing patterns and treatment outcomes for vertebral osteomyelitis for factors associated with adverse outcomes.

**Table 1.**
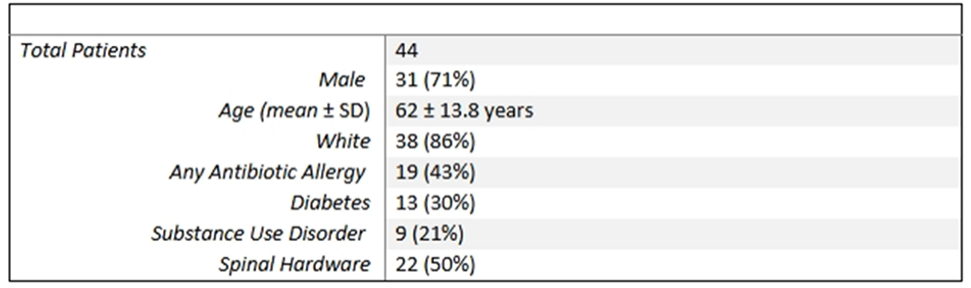
Study population of patients with vertebral osteomyelitis at Vanderbilt University Medical Center between July 1, 2020 and June 30, 2022.

**Table 2:**
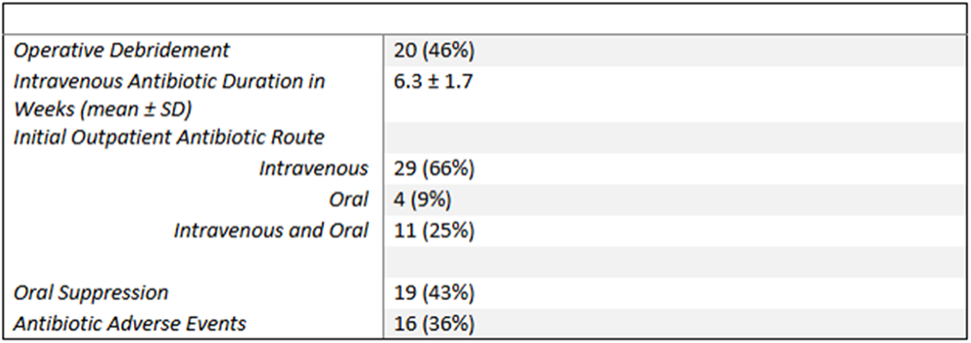
Treatment characteristics of patients with vertebral osteomyelitis at Vanderbilt University Medical Center between July 1, 2020 and June 30, 2022.

**Methods:**

Patients with vertebral osteomyelitis or spinal hardware infections hospitalized between July 1, 2020 and June 30, 2022 were identified by programmatic electronic health record data extraction. This period was chosen to allow for ≥24 months of follow up data. Exclusion criteria included concurrent diagnosis of sacral pressure ulcer and patients without at least one outpatient infectious diseases follow up appointment. Demographic factors, duration and route of antimicrobial therapy, and treatment outcomes were identified via chart review.

**Figure 1.**
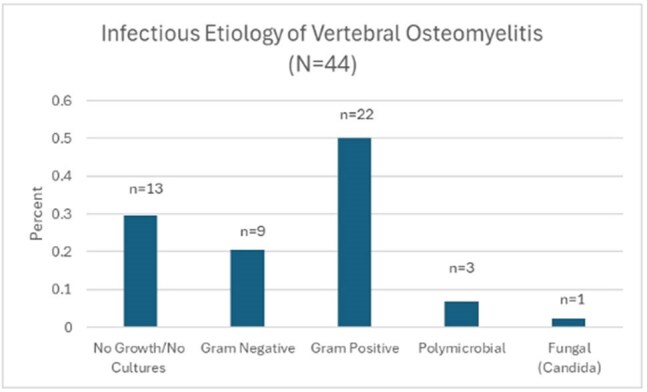
Infectious etiologies of vertebral osteomyelitis diagnoses at Vanderbilt University Medical Center between July 1, 2020 and June 30, 2022.

**Figure 2.**
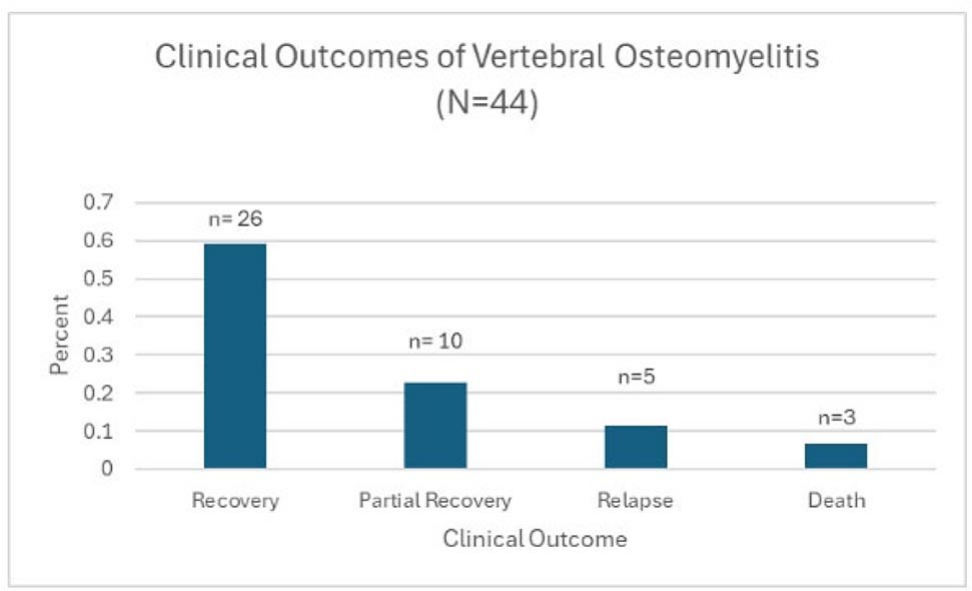
Clinical outcomes of vertebral osteomyelitis at Vanderbilt University Medical Center diagnosed between July 1, 2020 and June 30, 2022.

Recovery: survival; resolution of active infectious signs/symptoms; no clinically significant residual disability; Partial Recovery: survival; resolution of active infectious signs/symptoms; clinically residual disability; Relapse: survival; recurrence of active infectious signs/symptoms after apparent recovery or partial recovery; Death: death from any cause during study period

**Results:**

Of 64 patients identified via programmatic data extraction, 44 (69%) met inclusion criteria (Table 1), of whom 31 (71%) had a probable or confirmed infectious organism identified. Gram-positive organisms were identified in 22 (50%) cases (Figure 1). Twenty (46%) patients underwent operative debridement (Table 2). IV antibiotics were used in 40 (91%) initial outpatient antibiotic regimens. Mean duration of antibiotic therapy was 6.3 (±1.7) weeks, with a median of 6 weeks. 19 (43%) patients were transitioned to suppressive antibiotic therapy. Although 26 (59%) recovered, 5 (11%) relapsed and 3 (7%) died (Figure 2).

**Conclusion:**

These results suggest most patients at our institution with vertebral osteomyelitis receive just over six weeks of initial antibiotic therapy, concordant with current guideline recommendations. Despite emerging data of the non-inferiority of oral antibiotics, almost all patients still received intravenous antibiotic therapy with their initial regimen. Future steps include further exploration of patient factors associated with adverse clinical outcomes.

**Disclosures:**

Kaitlyn Reasoner, MD, Kindle Direct Publishing (Amazon): Royalties from a self-published book unrelated to topic of abstract Byron Stephens, MD, Globus: Grant/Research Support|Stryker: Grant/Research Support Milner Staub, MD, MPH, Eli Lilly: Stocks/Bonds (Public Company)|Gilead: Stocks/Bonds (Public Company)|Johnson & Johnson: Stocks/Bonds (Public Company)

